# Neuroendocrine crosstalk between sex and metabolic hormones: mechanisms and implications across the female reproductive spectrum

**DOI:** 10.1007/s44417-026-00014-7

**Published:** 2026-02-04

**Authors:** Virginie Goulet, Dali Léveillé, Jimeng Li, Alexandre Fisette

**Affiliations:** Research group in cellular signaling, Department of Medical Biology, Université du Québec à Trois-Rivières, Québec, Canada

**Keywords:** Hormones, Neuroendocrinology, Menopause, Reproduction, Leptin, Ghrelin, Adiponectin, Insulin, Estradiol, GLP-1, Diabetes, Obesity

## Abstract

Energy metabolism and fertility are intricately linked across the female lifespan, from puberty through pregnancy, lactation, and menopause, ensuring that nutrition aligns with reproductive demands. We review here the nature of the synergistic crosstalk between sex hormones (notably estradiol) and metabolic hormones (including insulin, leptin, adiponectin, GLP‑1, ghrelin) within the brain, across the female reproductive spectrum. Estradiol amplifies metabolic signaling via shared pathways such as PI3K/Akt and JAK/STAT and enhances receptor sensitivity and secretion of multiple metabolic hormones, supporting the regulation of appetite, energy expenditure, and glucose homeostasis. Menopause disrupts this integrated network as estradiol declines, resulting in metabolic imbalances characterized by impaired hormone sensitivity, weight gain, and insulin resistance. In contrast, pregnancy enhances hormonal crosstalk through placental hormones, triggering metabolic realignments necessary for fetal energy demands. However, excessive or dysregulated adaptations may contribute to disorders like gestational diabetes. Understanding these synergies, and how estrogen receptor-associated co‑transcription factors can modulate them, represents a promising therapeutic direction to restore metabolic and reproductive health during hormonal transitions such as menopause and pregnancy.

## Introduction

Fertility and energy metabolism are naturally intertwined throughout life, from sexual development to pregnancy, lactation and menopause, to coordinate feeding behavior with the vastly fluctuating energy needs stemming from reproductive processes. In addition, precise and coordinated substrate partitioning is an essential aspect of pregnancy, as the energy consumed by a single organism must be efficiently separated among many, to promote development and ensure survival. These metabolic realignments, spanning the entire reproductive spectrum, are largely triggered by coordinated changes in hormones secretion and sensitivities.

Hormones are essential chemical messengers for the survival and reproduction of living beings. They coordinate a wide range of biological functions such as metabolism, growth, and fertility [1]. However, beyond their individual roles, hormones also have the capacity to act synergistically, that is, to collaborate in a way that produces a physiological effect greater than the sum of their individual actions [2], allowing the organism to adapt efficiently to its needs.

## Sex and metabolic hormones

Among the hormones that interact synergistically, metabolic hormones (mainly insulin, leptin, adiponectin, glucagon-like peptide-1 (GLP-1), and ghrelin) and sex hormones (mainly estrogens, progesterone, and androgens) stand out. These two hormonal groups converge at the hypothalamic-pituitary-gonadal axis in the brain to integrate key information about the organism’s energy status and regulate reproduction [1, 3].

To illustrate this synergy, we will consider the example of leptin. This metabolic hormone informs the brain about the level of energy reserves [4, 5] and, if these reserves are sufficient, leptin indirectly stimulates the secretion of gonadotropin-releasing hormone (GnRH), a master hormone of fertility [6]. Thus, reproductive system activation occurs only when metabolic signals allow it, ensuring that the energy-demanding function of reproduction is only initiated when the body has sufficient resources [7].

Sex hormones, for their part, do not only play a key role in reproduction but also modulate metabolism. For instance, estrogens, mainly female hormones, play an important role in regulating sugar and fat metabolism, influencing insulin sensitivity and body fat distribution [8].

Therefore, metabolism and reproduction do not operate in isolation. Quite the opposite, hormonal synergy ensures a finely tuned coordination of these physiological systems, prioritizing the organism’s needs based on available resources. Despite their potential synergy, metabolic and sex hormones have distinct origins and mechanisms that must be further explored.

### Origins of key metabolic hormones and their signaling pathways

Among the most frequently studied metabolic hormones, due to their importance in regulating metabolism and their pharmacological relevance, we will focus here on insulin, leptin, adiponectin, GLP-1, and ghrelin.

#### Insulin, a regulator of blood glucose

After being secreted by the β-cells of the pancreatic islets of Langerhans [9], insulin regulates blood glucose by facilitating glucose uptake into skeletal muscle, liver and adipose tissue [10, 11] and by inhibiting hepatic glucose production [10]. Insulin also stimulates glycogen and lipid synthesis in these target organs [12]. After a meal, insulin binds to insulin receptors (IR) and activates the IRS-PI3K-Akt-FoxO signaling pathway [8]. Then, through Akt, Glucose Transporters Type 4 (GLUT4) are translocated to the plasma membrane, allowing glucose entry into cells. During fasting, this mechanism is less active due to low insulin levels in the bloodstream [13]. Insulin also plays a central role in reducing appetite [13], as it binds to IR on energy-balance-regulating neurons such as anorexigenic pro-opiomelanocortin (POMC) neurons, which promote a negative energy balance [14, 15]. It also inhibits orexigenic neurons expressing neuropeptide Y/agouti-related peptide (NPY/AgRP), that promote a positive energy balance [14, 15].

#### Leptin, a messenger of energy reserves

Leptin is mainly produced by adipocytes in correlation to the size of adipose tissue, informing the hypothalamus of the available energy stores of the body [4, 5]. In the short term, its secretion is moderately modulated: it decreases after a short fasting period and increases several hours after a meal [16–18]. By binding to the long-form leptin receptor (Ob-Rb), which is abundantly expressed in the arcuate nucleus of the hypothalamus [19, 20], leptin exerts an anorexigenic effect [20]. Specifically, it stimulates anorexigenic POMC neurons and inhibits orexigenic NPY/AgRP neurons, thus reducing appetite and increasing energy expenditure [20]. This binding triggers the JAK/STAT signaling pathway [21], leading to the recruitment and phosphorylation of the STAT3 protein [22]. Phosphorylated cytoplasmic STAT3 molecules dimerize and translocate to the nucleus to regulate gene expression at specific genomic regions, notably the promoter region of the POMC gene, which promotes satiety [22, 23].

#### Adiponectin, the insulin-sensitizing hormone

Like leptin, adiponectin is predominantly secreted by adipocytes [24]. However, in contrast to leptin, its circulating levels are inversely proportional to the total amount of body fat mass [24, 25]. Thus, in the context of obesity or insulin resistance, adiponectin concentrations are markedly decreased, which increases the risk of developing type 2 diabetes and even cardiovascular diseases, given the well-established role of adiponectin in vascular protection [25–27]. Under normal physiological conditions, adiponectin plays a major key role in maintaining metabolic homeostasis by sensitizing to insulin, leading to an increase in fatty acid oxidation and glucose utilization in skeletal muscle, while inhibiting hepatic glucose production [28]. To exert these peripheral effects, adiponectin binds to its receptors AdipoR1 and AdipoR2, which activate the AMPK and PPARα signaling pathways [29]. Beyond their peripheral presence, these receptors are also present in the central nervous system, particularly in the arcuate nucleus of the hypothalamus, where they activate POMC neurons while inhibiting AgRP/NPY neurons. In opposite, during fasting, adiponectin activates hypothalamic AMPK, which leads to increased food intake [30]. In contrast, under conditions of energy abundance, adiponectin improves insulin and leptin sensitivity [31].

#### GLP-1, a mediator of postprandial satiety

In its effort to avoid excessive energy intake, the body produces another important anorexigenic hormone: glucagon-like peptide-1 (GLP-1). It is secreted by L-cells of the intestinal epithelium in response to the presence of nutrients, particularly glucose, fatty acids, and amino acids [32]. Hence, its secretion increases rapidly after a meal but is almost absent during fasting [33]. Interestingly, the secretion peak varies with meal composition; for example, high-fat meals induce a stronger GLP-1 response [34]. Moreover, metabolic status affects secretion: individuals with obesity or type 2 diabetes show reduced GLP-1 secretion compared to healthy individuals [35]. GLP-1 stimulates insulin secretion by binding to the GLP-1 receptor (GLP-1R) on pancreatic β-cells, activating the adenylate cyclase (AC)-cAMP signaling pathway and second messengers like PKA and Epac2 [36]. This pathway regulates insulin secretion when glucose levels are high [36]. GLP-1 also plays a major role in the central nervous system, particularly within the hypothalamic-pituitary-gonadal axis, acting on anorexigenic POMC neurons and directly contributing to postprandial appetite suppression [37]. Notably, GLP-1 receptors are found on a subpopulation of POMC neurons that express little or no leptin receptors, highlighting how apparently homogenous neuronal population still exhibit heterogeneity, with multiple neuronal subpopulation that respond to specific hormones [38].

#### Ghrelin, the appetite-stimulating hormone

Amid this symphony of anorexigenic hormones, ghrelin stands out for its opposing orexigenic role. It is primarily secreted by endocrine cells in the stomach lining in anticipation of a meal or in response to reduced caloric intake [39]. Its levels fall rapidly after eating, helping to suppress appetite [40]. Ghrelin signals the brain about energy deficits to stimulate appetite, notably by directly activating NPY/AgRP neurons and indirectly inhibiting POMC neuron activity [41, 42]. Additionally, it modulates the activity of certain dopaminergic neurons in the arcuate nucleus of the hypothalamus, though its central role in appetite mostly involves orexigenic neurons in this region [41]. This involves ghrelin binding to its receptor GHS-R1a, a G protein-coupled receptor (Gq, Gi/o, G13), which activates the PLC/IP3/Ca^2+^ signaling pathway [43].

### Origins of sex hormones and their signaling pathways: focus on estradiol

Following metabolic hormones, it is essential to examine sex hormones, given their major role in regulating energy metabolism. These steroid hormones are classified by their predominance in each sex: female sex hormones include estrogens (estradiol, estrone, estriol, and estetrol) and progesterone, while male sex hormones include androgens (testosterone and dihydrotestosterone—DHT) [44, 45].

While their roles in puberty and reproduction are well-known, it is important to highlight that their function in metabolism is also crucial. We will focus here on female sex hormones, particularly estradiol (E2), due to its protective effect against metabolic disorders such as obesity and type 2 diabetes [46]. This protection is thought to stem from evolutionary metabolic adaptations for gestation and lactation based on food availability [47].

#### Estradiol production and hormonal rhythmicity

E2 is mainly produced by the ovaries, but also by adipose tissue and the adrenal glands [48]. Its secretion rhythm varies based on physiological stage: during puberty, across the menstrual cycle, and at menopause [49].

Throughout the menstrual cycle, E2 increases during the follicular phase and reaches a peak just before ovulation [50]. A secondary, smaller peak occurs in the luteal phase, followed by a sharp decline if fertilization does not happen [50]. Daily variations in E2 are also observed, with peak levels in the morning [50, 51].

During puberty, hypothalamic gonadotropin-releasing hormone (GnRH) stimulates the pituitary secretion of follicle-stimulating hormone (FSH) and luteinizing hormone (LH), which trigger ovarian estrogen production, marking the onset of estradiol increase and the beginning of ovarian cycles [52]. In contrast, at menopause, ovarian E2 production drops drastically to very low but steady levels, insufficient to sustain menstrual cycles [53]. This acute hormonal drop is a unique phenomenon with no equivalent in males [54], making it a model for studying metabolic disorders linked to hormonal loss.

#### Signaling mechanisms

E2 acts mainly through two types of estrogen receptors: ERα and ERβ, each with multiple isoforms [55]. These receptors are found not only in reproductive organs but also in bone, liver, colon, skin, salivary glands, and the brain, especially the hypothalamic-pituitary-gonadal axis [56]. Both contribute to ovarian function, but ERα is more involved in metabolic regulation [57].

The classical E2 signaling pathway involves passive ligand diffusion into the cell and binding to cytoplasmic estrogen receptors [58]. The receptor-ligand complex dimerizes and translocates to the nucleus, where it acts as a transcription factor on target genes, mostly those with an estrogen response element sequence [58].

A less typical signaling mechanism occurs when E2 binds to membrane-bound receptors [58], triggering signaling cascades involving ion channel modulation, second messengers, and transcription factor activation like Sp-1 [58].

Estradiol also activates the PI3K, mTOR, and Sirt1 signaling pathways, all of which play critical roles in insulin sensitivity and the anorexigenic effects of estradiol [8, 48, 59]. ERα remains the main mediator of E2 activity, as its interaction is essential for initiating downstream signaling.

## Hormonal control of body weight

Energy balance is defined as the equilibrium between caloric intake and energy expenditure. With precise energy homeostasis regulation, body weight remains stable in healthy adult individuals. However, in a state of chronic positive energy balance, where caloric intake exceeds energy expenditure, weight gain occurs. As detailed in the previous section, hormones, as endocrine messengers, modulate energy balance and body weight. The hypothalamus, located in the ventral brain above the pituitary gland and below the third ventricle, is a major target for these hormones, and must integrate these multiple peripheral hormonal signals to produce a single, coherent response to orchestrate energy homeostasis, and therefore control body weight.

### Hormonal regulation of energy balance

In states of negative energy balance—such as fasting or caloric restriction—the hypothalamus integrates peripheral hormonal cues to restore homeostasis by promoting food intake and energy conservation. Ghrelin levels will rise, increasing hunger and meal initiation via their activation of AgRP neurons [60]. Concurrently, leptin and insulin levels drop, reducing their inhibitory tone on AgRP neurons and disinhibiting feeding behavior [61, 62]. Low leptin also diminishes activation of POMC neurons, thus their anorexigenic effects [63]. Insulin signaling in the hypothalamus, diminished during fasting, results in reduced energy expenditure and promotes hepatic glucose production [10]. Meanwhile, GLP-1 secretion is suppressed, minimizing its anorexigenic and insulinotropic roles, aligning with the physiological drive to increase energy intake and preserve glucose.

During chronic positive energy balance—such as in overfeeding or obesity- insulin and leptin levels are persistently elevated [64], signaling energy surplus to the hypothalamus [4, 42]. However, prolonged exposure induces central leptin and insulin resistance, particularly in the hypothalamus, blunting their ability to activate POMC neurons or inhibit AgRP neurons [65, 66]. This disrupts appetite control and energy expenditure regulation, perpetuating excess intake and fat accumulation. Adiponectin levels are in turn also downregulated, which further promotes whole-body insulin resistance [31]. GLP-1, normally enhancing satiety and insulin secretion [36, 37], may be upregulated postprandially, but its long-term effectiveness is eventually reduced with the desensitization of GLP-1 receptors [67]. Conversely, ghrelin levels are suppressed in obesity, yet its orexigenic drive may persist due to impaired central feedback inhibition [68]. Together, this hormonal profile promotes a maladaptive state in which hypothalamic circuits fail to adequately restrain energy intake or increase expenditure, reinforcing metabolic dysregulation. A crucial aspect here is linked with altered hormonal sensitivity, as chronic energy surplus diminishes the sensitivity to metabolic cue, including insulin, leptin or GLP-1, while preserving abnormally high signaling of ghrelin.

### Estrogens: how they synergize with metabolic hormones

As evidenced in the previous section, the sensitivity of the organism—particularly the brain- to metabolic hormones is paramount for efficient energy homeostasis. Chronic positive energy balance, which leads to weight gain and obesity in the long term, is both a cause and a consequence of reduced sensitivity to hormones including leptin, insulin and GLP-1, and exacerbated sensitivity to ghrelin, as we described. While this phenomenon is consistently observed in male mammals, females were shown to be partially protected against metabolic disorders (including obesity and type 2 diabetes) during reproductive life, while showing increased sensitivity to these diseases after menopause, independent of aging [69]. Importantly, this appears to be linked with increased sensitivity in the brain to various metabolic hormones, suggesting a synergistic effect with female sex hormones.

#### Estrogen, insulin & glucose metabolism

At physiological levels typical of the premenopausal phase in women, estrogen generally has a protective effect against diabetes by enhancing insulin sensitivity and lowering blood glucose levels [8]. This appears to be partly mediated via the convergence of E2 and insulin (and IGF-1) signaling pathways toward PI3K/Akt cascade in the brain [49]. Co-stimulation leads to stronger pathway activation than either hormone alone, indicative of a functional synergy [70, 71]. However, hyperestrogenic states—such as those observed in some cases of gestational diabetes, polycystic ovary syndrome and ovarian hyperstimulation syndrome—can lead to insulin resistance [72]. One proposed mechanism is that estrogen binds directly to insulin and its receptors, interfering with insulin signaling and thereby inducing insulin resistance [72]. In both cases, this showcases the ability of estrogen to modulate insulin sensitivity significantly, in an estrogen levels-dependant fashion.

#### Leptin signaling & sex hormones

Several studies have shown that E2 exerts effects in the hypothalamus similar to those of leptin [73, 74]. For instance, E2 can regulate synaptic plasticity in the arcuate nucleus and reduce body weight in ob/ob mice, mimicking the outcomes of leptin treatment [75]. Moreover, estrogen deficiency has been shown to contribute to central leptin resistance and increased adiposity [65]. Conversely, estrogen administration in both rats and humans significantly increases serum leptin levels [76].

The similarities between E2 and leptin effects on metabolism are not fortuitous; E2 exerts a major part of its protective actions by sensitizing hypothalamic neurons to leptin. A recent publication identified the role of Cited1 within POMC neurons in this interaction, as Cited1 allowed a synergistic transcriptional outcome via the formation of a Cited1–ERα–STAT3 complex, which then mediates changes in key aspects of feeding behavior and obesity regulation [77].

#### Adiponectin and estradiol

It has been suggested that adiponectin and sex hormones such as estradiol can interact, indicating that hormonal variations throughout the female reproductive cycle may influence adiponectin’s metabolic actions on the organism [78]. Clinical studies have demonstrated that serum adiponectin levels have a negative correlation with free E2 in menopause [79]. Similar results can be found in hormone replacement therapy, in which it was reported that supplementation of E2 leads to lower plasma adiponectin concentrations [80]. Similarly, exposure to bisphenol-A, which can mimic E2, leads to a decline of adiponectin levels [81]. However, low adiponectin was detected in ERα-KO mice [82]. Moreover, adiponectin-deficient mice present a range of reproductive disorder phenotypes, as E2 level at diestrus is lower than controls [83]. This discrepancy between the inverse correlation of adiponectin and E2 levels could be linked with the pro-inflammatory phenotype observed in the adipose tissue of these models, which could in turn reduce adiponectin secretion [84, 85]. Overall, the interplay between adiponectin and E2 remains incompletely understood, but typically manifests as an inverse correlation.

#### GLP-1, estradiol and the efficacy of incretin therapies

Compelling evidence also supports the pronounced and extensive synergistic effects between estrogen and GLP-1. Experimental studies, conducted both in vitro and in vivo, reveal that estrogen directly stimulates GLP-1 secretion from intestinal L cells, which is specifically dependent on the presence of ER β [86]. Estrogen also potentiates the anorectic responses elicited by central GLP-1 receptor activation, as well as reward behavior [87]. Further mechanistic insights indicate that the combined action of E2 and GLP-1 reduces feeding by synergistically activating neurons in multiple regions, including the paraventricular hypothalamus (PVN) and the supramammillary nucleus [86, 88, 89]. The mechanistic underpinnings behind these effects remain however relatively unclear. In support of these preclinical findings, GLP-1 based incretin therapies are generally more efficient in human females than in males [90, 91], but this difference is lost after menopause [92]. These findings have paved the way for innovative pharmacological interventions, notably a dual agonist combining GLP-1 and E2, which demonstrated superior glucose-lowering capabilities in diabetic murine models when compared to GLP-1 monotherapy [93].

#### Ghrelin and an inconclusive link with sex hormones

Evidence for estrogen and ghrelin synergy is complex and sometimes inconsistent. While E2 has been shown to increase the effects of metabolic hormones such as insulin, leptin or GLP-1, it is important to remember that ghrelin exerts opposite, orexigenic effects. A first effect of estradiol appears to be a lowering of ghrelin levels, as animals deficient in E2 exhibit elevated plasma ghrelin concentrations [94]. Conversely, a separate clinical investigation yielded a divergent outcome, reporting an increase in plasma active ghrelin levels following estrogen supplementation in postmenopausal women [95]. Smith et al. speculated that the test was confounded by total, des-acyl or ghrelin, especially des-acyl ghrelin is often considered inactive [96]. In addition to regulating ghrelin levels, estradiol, much like with other metabolic hormones, also appears to synergistically regulate the outcome of ghrelin effects. While estradiol increased the anorectic potency of insulin, leptin or GLP-1, it was reported to decrease the orexigenic potency of ghrelin in preclinical models [97]. This is counterintuitively accomplished partly via estradiol-dependent *increases* in ghrelin responsiveness, but in a particular subset of neurons, which express kisspeptin [98]. These neurons, upon activation, usually promote a reduction in food intake, in opposite to AgRP neurons, while both express ghrelin receptors. Thus, despite boosting ghrelin receptor availability, estradiol globally reduces ghrelin-induced hunger.

#### Estradiol signaling: a complex route with multiple players

These findings highlight an important aspect of E2 actions; indeed, E2 signaling is highly complex, largely due to the involvement of numerous co-transcription factors that modulate estrogen receptor activity in a context-dependent manner [99]. These co-regulators, such as Cited1, can integrate signals from multiple hormonal pathways, enabling precise control over gene expression and cellular response. Through this mechanism, co-factors not only influence the intensity and specificity of estradiol effects, but could also mediate hormonal synergies, such as those between estradiol and leptin, insulin, GLP-1 or ghrelin, fine-tuning the metabolic outcomes in a sex hormone-dependent way. This complexity is especially relevant in tissues like the hypothalamus, where metabolic and reproductive signaling must be tightly coordinated.

### A protective role for sex hormones—the case of menopause

Menopause (also known as reproductive senescence) typically occurs in women between the ages of 45 and 55, and the transition is generally divided into three stages: perimenopause, menopause, and post menopause. In humans, menopause is believed to be triggered by ovarian follicle depletion [100] and is associated with hormonal shifts and imbalances [101]. Hormonal perturbations are characterized by decreased levels of estrogen and progesterone, and increased levels of FSH and LH. In contrast, mice exhibit a different hormonal profile during reproductive aging, showing slight increases in estrogen and progesterone levels [102]. As a result, chemical-induced ovarian atresia (via 4-vinylcyclohexene diepoxide treatment) and surgical (ovariectomy, OVX) models are commonly used to mimic human menopause in rodent studies [102].

However, during the hormonal fluctuations of menopause, previously described hormonal synergies -such as those involving estrogen, leptin, adiponectin, insulin, GLP-1 and ghrelin- are disrupted. As estrogen levels decline, resistance to both leptin and insulin often emerges [103, 104]. An example of the proposed underlying mechanism involves impaired activation of hypothalamic STAT3 signaling [77], potentially involving Cited1 as well. Other teams have shown that OVX mice developed glucose intolerance and insulin resistance, while both central and peripheral administration of E2 can reverse the effect, potentially due to estrogen’s enhancement of POMC neuron excitability in the hypothalamus [105, 106]. Similarly, peripheral or central administration of E2 to ovariectomized (OVX) rats restores leptin sensitivity in the hypothalamus [107]. Thus, physiologically, the loss of this synergistic effect of estradiol with metabolic hormones is linked with heightened risks of developing metabolic diseases, including obesity and type 2 diabetes [108–110]. In support of this causal inference, hormonal replacement therapy in postmenopausal woman is associated with metabolic profile improvements, particularly for glucose handling and visceral adiposity [111, 112]. Surprisingly, as mentioned previously, ovarian dysfunction and diminishing E2 levels rather increase adiponectin concentrations in humans and rats, which may partly mitigate the adverse metabolic phenotype observed [113].

While prolonged hormonal therapy is likely not an option to protect postmenopausal woman against metabolic diseases in the long term, a promising angle of research is that of estrogen receptor co-activators, which could possibly be taken advantage of to restore metabolic hormone sensitivity. This could allow specifically the promotion of satiety and metabolic homeostasis without the widespread and unwanted effects of estrogens on the body.

## Pregnancy: a temporary hormonal paradigm

Pregnancy is a unique metabolic state requiring effective regulation of behavior and metabolism such as food intake, energy expenditure [114–117] and fat deposition [118, 119]. The maternal body needs to adapt to establish a favorable and balanced environment for embryonic implantation, development and promote fetal growth, all while establishing a positive energy balance [115, 120, 121] for storage of resources for the demands of lactation [114, 117, 118, 122]. All these adjustments must be tightly monitored as the slight fluctuation can inflict consequences on both the mother and fetus [123, 124]. Dramatic changes are made in the endocrine system with remarkable fluctuations on hormones to ensure the best environment for the fetus and thus a successful pregnancy [114]. Furthermore, adequate metabolic homeostasis needs to be maintained for reproductive health [125] since both energy deficiency and hyper caloric intake can result in poor fertility [126, 127]. Thus, both hormonal and metabolic adjustments are required to warrant an optimal pregnancy.

### Metabolic realignment during pregnancy

Pregnancy requires a wide variation of metabolic changes to promote fetal growth [114, 122]. Pregnancy has a diabetogenic effect on the metabolism [124]. Starting at mid-pregnancy, the hormones secreted from the placenta take over the maternal metabolism and physiology to implement a state of insulin resistance, thus reducing insulin sensitivity [124, 128]. This condition of insulin resistance is critical to maintain nutrient intake of the fetus [124]. Pregnancy can be separated into two distinctive phases, the first being characterized as the “maternal anabolic phase” in early pregnancy, in which there is an increase in energy reserve in the maternal body and the second known as the “maternal catabolic phase”, later in pregnancy where there is a reduction of insulin sensitivity and an increase in maternal glucose concentration [116] to support fetus growth and development [122, 124, 129, 130]. The glucose metabolism adapts in these two phases to accommodate the energy requirements of both the maternal body and the fetus via changes in the pancreas. In fact, the pancreas increases the mass and function of the β-cells to stabilise the high levels of glucose found in circulation and to even out the insulin resistant state occurring during pregnancy [114, 131]. In humans, the fetus is unable to perform glucogenesis, and thus relying solely on the glucose supply of the mother and the placenta [132, 133]. As such, around the 26th week of pregnancy, when the fetal demands in glucose increase, maternal glucose homeostasis shows a delay in blood glucose clearance [134] and upregulated the hepatic glucogenesis [135], induced by the action of multiple hormones, notably estrogen [136]. There is also an impaired response to insulin in peripheral tissues such as skeletal muscle, and a decrease in hepatic suppression of glucogenesis [124, 129]. Consequently, more circulating glucose is supplied to the fetus and placenta [137].

Pregnancy also requires changes in the lipid metabolism that are accompanied by modifications in the function and morphology of the adipocytes, such as an increase in the number of insulin receptors, to accommodate the fat storage needed during the first and second trimester of the pregnancy [122, 138], both known to be anabolic phase of gestation which favor lipid deposition and inhibit lipolysis [124, 132, 139]. In the third trimester a change happens in the regulation of the lipid metabolism: it shifts from an anabolic state to a catabolic one, therefore limiting fat deposition, making lipids the main source of energy for the maternal body, thus keeping glucose for the fetus [124, 139].

### Pregnancy reprograms the hormonal communication system

As the maternal body faces a challenge trying to provide for itself and the growing demands of the fetus, it adapts and henceforth stays in a state of positive energy balance [114, 115, 120, 121]. To achieve this evolutive and major metabolic realignment during the various phases of pregnancy, a dynamic and complex hormonal signature is at play. These hormones transmit the necessary information about the mother’s metabolic homeostasis to the brain, mainly the hypothalamus, to finely tune processes which include appetite and satiety [114, 115, 123]. These adjustments are necessary to maintain a state of positive energy balance. In addition, the sensitivity to some hormones is also altered, which altogether induces a new metabolic regulation to ensure successful pregnancy as well as survival. This section will introduce the main endocrine adjustments required during pregnancy.

### Sex hormones and a new source of messengers, the placental hormones

**Fig. 1:**
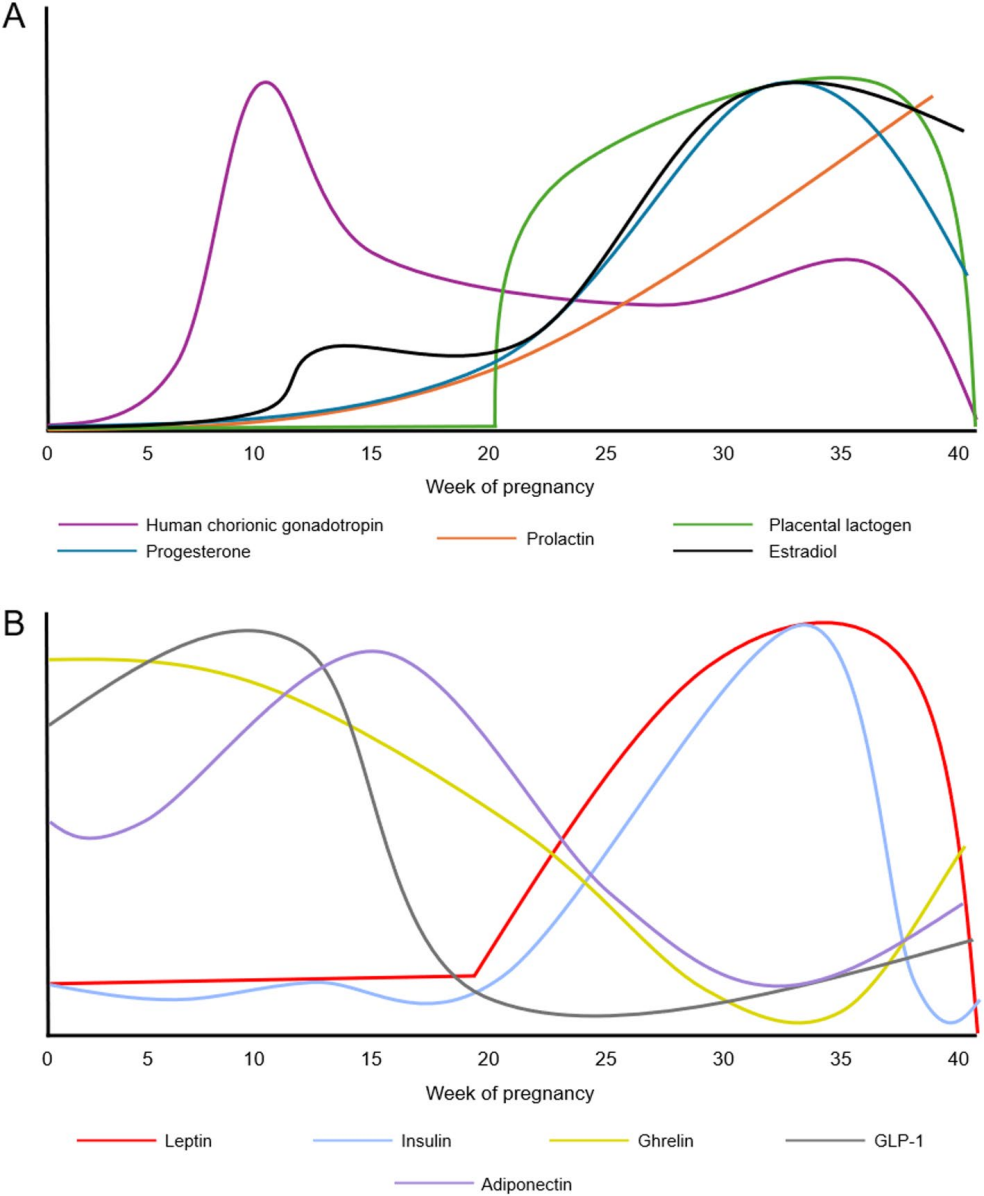
Hormonal fluctuations during pregnancy. Simplified representation of the relative concentration of sex and placental hormones (**A**) and of traditional metabolic hormones (**B**), some of which also have placental sources, during human pregnancy

In addition to fluctuation in traditional sex hormones, multiple crucial new hormones are introduced during pregnancy and lactation (illustrated in Fig. 1a), to promote and maintain the ideal environment and the optimal pregnancy [132, 140].

#### Estradiol and progesterone

Both estradiol and progesterone prepare the endometrium for embryonic implantation [141]. In pregnant mice, the expression of estradiol is reduced in the beginning as its stimulating effects on satiety neurons and inhibitory effects on appetite neurons would be counterproductive, and as it could have nefarious effects on the embryonic development by disrupting its implantation in the endometrium [114]. The increase in appetite in early pregnancy could be linked to the lack of the anorectic effect of estradiol while having a simultaneous increase in progesterone levels [114, 117]. Progesterone secretion rise alongside food intake and has been hypothesized to have a role in the increased food intake seen during gestation [142], in addition to its crucial role in ensuring safe implantation of the fetus in the uterus [114].

#### Placental growth hormone (PGH)

In contrast to humans, most mammalian species do not produce placental growth hormone (PGH) [123, 143, 144]. In fact, murine placenta expresses placental-specific genes related to prolactin, such as placental lactogens (PL), rather than growth hormone [123, 143, 144]. To compensate for this, levels of growth hormone from the pituitary increase during pregnancy [145, 146]. PGH is very similar to human growth hormone. It is in fact one of its variants, but produced by the placenta [132, 147]. PGH is expressed in the syncytiotrophoblast and secreted from placenta in the maternal circulation [132, 148] slowly replacing the human growth hormone, secreted from the pituitary, as the pregnancy goes on [123, 132, 147, 149–151]. PGH plays a role in adjusting the maternal metabolism by regulating glucogenesis and lipolysis [132, 152]. PGH also acts as an insulin antagonist, playing a role in insulin resistance by directly adjusting the maternal insulin-like growth factor (IGF-1) [132, 147, 148, 153, 154]. PGH also stimulates trophoblast invasion [155] by acting in an autocrine and paracrine manner [132, 147, 156] and placental growth by acting on its receptor on the syncytiotrophoblast [157].

#### Human placental lactogens (hPL)

Human placental lactogen (hPL), also known under the name chorionic somatotrophin hormone, is a type of growth hormone [132] detectable in the umbilical cord blood and the maternal blood at the sixth week of pregnancy [147, 158]. hPL acts by binding prolactin receptors and as the pregnancy progresses, the maternal prolactin levels decline making hPL the dominant lactogenic hormone [147]. hPL has several roles through the pregnancy such as metabolic regulation by promoting insulin resistance and thus increasing the maternal glucose levels while decreasing its usage and promoting lipolysis to supply the fetus [132, 147, 158, 159]. hPL also influences maternal β-cell by increasing their mass and function, hence stimulating their adjustment to the increased insulin requirement needed during pregnancy [158, 160]. With estrogen, progesterone and prolactin, hPL helps develop the necessary mammary ductal and alveolar growth for lactogenesis [158].

#### Human chorionic gonadotropin (hCG)

hCG is a hormone produced by the syncytiotrophoblast detected in the maternal blood roughly around a week after fertilization [147, 161]. Its concentration increases during the first trimester [147]. Among multiple other functions, hCG has a luteotropic effect, meaning it acts on the maternal corpus luteus to maintain the production of progesterone in the ovaries [162], consequently maintaining the endometrium environment [147, 161, 163, 164]. In early pregnancy, hCG plays a crucial role in regulating the hemochorial placentation, meaning it regulates the uterine, fetal and placental growth while also protecting the pregnancy from immune rejection [147, 161].

### Sex & metabolic hormones in pregnancy: old players with new roles

While multiple organs are implicated in whole-body energy balance and substrate partitioning during pregnancy, we will focus here on the neuroendocrine basis of metabolic realignment and how metabolic hormones concentration and signaling are altered in the hypothalamus (illustrated in Fig. 1b).

#### Insulin

During pregnancy, the mother provides the glucose as the main energy source to support the needs of the growing fetus and placenta [114, 116]. Since the glucose is passively transferred from the maternal circulation to the foetus and placenta [114, 116, 165] the mother needs to keep a higher basal concentration of glucose than the needs of the fetus, which as pregnancy progresses becomes a challenging event [116]. Hence, to overcome this, the maternal peripheral tissues, such as skeletal muscle, become insulin resistant to prevent their uptake of glucose, sparing it for the fetus [116, 124, 129]. There is also an increase in the secretion of insulin from the β-cell due to their pregnancy-specific expansion in mass and their decreased threshold for glucose-stimulated insulin secretion [116, 123, 131].

Some of the possible mechanisms regulating the new insulin resistant state during pregnancy involve an increase in the transport of insulin to the brain across the choroid plexus and into the cerebrospinal fluid [166, 167], and an elevated level of degradation of insulin in the parenchyma [168]. The parenchymal degradation may be due to an increase of the insulin-degrading enzyme (IDE) that could be increased during pregnancy [166], as in vitro studies showed an elevated level of IDE in neurons when treated with insulin [169]. Another mechanism may be an attenuated signalling in the brain through the PI3K pathway used by insulin [170]. During the insulin resistant state in pregnancy, there is a downregulation of the phosphorylation of the insulin receptor [171]. Adding up to all this, there may also be a central insulin resistance during pregnancy in certain specific regions of the hypothalamus, such as the arcuate nucleus, a major site of action of insulin, therefore altering transiently the brain sensitivity to that specific hormone [172].

#### Leptin

During pregnancy, an increase in circulating leptin can be observed [114, 173, 174]. This increase could be due by the accumulation of adipose tissue coupled with placental production of leptin [116, 123]. Despite this hyperleptinemia, appetite in pregnant women does not decrease, highlighting the hyperphagia-induced state of pregnancy and pointing towards a state of leptin resistance [114, 115, 117, 123, 173, 175, 176]. In humans, leptin levels and hyperphagia increase concomitantly throughout gestation [132], but there is an obvious increase during the second trimester [114, 177]. In mice, leptin concentration increases progressively throughout the pregnancy up until the second week where it peaks and lasts until the end of pregnancy [114, 173, 174].

Several mechanisms have been proposed to explain the leptin resistance state of pregnancy, including the reduced transport of leptin in the brain [114, 116, 173, 175, 176] and negative feedback of the downstream signaling pathway of leptin (JAK/STAT) [114, 116, 117, 175, 178]. Suppression of leptin transport in the brain could be due to a reduced activity of the saturable transporter [179] or to an increase in the expression of leptin-binding protein derived from the placenta which prevents the uptake by the brain [180–182]. The latter mechanism stems from the reduced action of leptin in phosphorylating the factor STAT3 in its active form rendering it inactive in the pathway thus reducing the response altogether [114, 116, 117, 175, 178]. Whether estradiol co-transcription factors are at play in pregnancy-induced leptin resistance remains unknown.

#### Adiponectin

In normal pregnancy, circulating maternal adiponectin levels progressively decline with advancing gestation [183–186]. Adiponectin levels during the first trimester do not differ from those of non-pregnant individuals and may increase during early pregnancy [185, 187]. In contrast, adiponectin levels decrease during the third trimester, coinciding with reduced maternal insulin sensitivity [184, 186, 188]. This hypoadiponectinemia may contribute to the progressive decline of insulin sensitivity observed in late gestation [183, 184]. Consistent with its role in insulin sensitivity, low adiponectin levels in early pregnancy could be an indicator of gestational diabetes mellitus (GDM) and type 2 diabetes [187, 189–192]. Adiponectin may also participate in implantation, as both adiponectin receptors are expressed in the endometrium [193], and reduced receptor expression is correlated with recurrent implantation failure [194].

#### Glucagon-like peptide 1 (GLP-1)

A study done in mice lacking GLP-1 showed that around half of the embryos were resorbed in the second part of gestation compared to control mice, highlighting the crucial role of GLP-1 in reproduction [195]. GLP-1 might modulate the activity of the GnRH neurons found in the hypothalamus [196–199], which are crucial regulators of the reproductive system [126, 200, 201]. When the body sense the potential conception, there is an increase in GLP-1 circulation levels coupled with an increased hypothalamic responsiveness to GLP-1 which could indicate that the body is ready to take on the challenge of pregnancy as soon as the metabolic homeostasis is optimal [125]. Whether this is linked with estradiol changing levels remains unknown.

#### Ghrelin

In rodent pregnancy, ghrelin is hypothesized to be a key factor necessary for successful embryo implantation [202, 203]. This could be because its inhibition during the peri-implantation phase impairs the capacity of the embryo to successfully attach, hence increasing embryo loss [204, 205]. Some studies hypothesize that the increase in ghrelin seen in early pregnancy [206–208] could be a factor in the endometrial decidualization, a process playing important roles in the protection of the embryo from immune rejection and the embryonic development preceding the formation of the placenta [209–211] and the formation of the placenta [212, 213]. Ghrelin levels keep on increasing in mid-pregnancy and decrease rapidly in late gestation when the full-term human placenta is formed [132, 208, 214]. This fluctuation in ghrelin levels parallels the homeostatic changes in the mother, characterized by increased food intake and weight gain, consistent with the orexigenic actions of this hormone [214].

There are coordinated changes in hormonal levels across the different stages of pregnancy, to ensure the most appropriate metabolic regulation. In early pregnancy, up until mid-pregnancy, the levels of insulin are still relatively low, while ghrelin levels rise [207]. On the other hand, during late pregnancy, when the maternal body is in a state of hyperinsulinemia and hyperleptinemia, the levels of ghrelin drop [215]. This highlights the potential effects of insulin on ghrelin secretion [207, 214].

#### Prolactin

Prolactin is a hormone produced and secreted by lactotrophs, specialized cells, found in the anterior pituitary [216–218]. It is part of a larger group of hormones known as “lactogenic hormones” also including PL and growth hormones in humans [219]. Its main role is milk production [216] as well as the mammary gland development [218]. In a non-pregnant state, prolactin levels are maintained at a low basal due to a negative feedback loop [218, 219].

During pregnancy, in humans, concentration of both prolactin and placental lactogen rise slowly throughout pregnancy [114, 220]. On the other hand, there is a unique secretion pattern of prolactin in rodents. In fact, the mating stimulus induces a prolactin production in the form a of a surge twice daily during the early and up to mid pregnancy [114, 115, 219, 220]. This highlights the presence of high concentration of prolactin at the onset of pregnancy [221]. Despite these surges, in the second half of the pregnancy, PL becomes the main lactogenic hormone in circulation in the maternal blood [219]. Since PL and prolactin act on the same receptor, it thereby inhibits the prolactin secretion from the pituitary [222] and bypasses the negative feedback loop and henceforth provides the prolactin receptor activation until delivery [115, 219, 222]. Strong evidence suggests that prolactin could have a role in the energy metabolism, specifically in the positive energy balance observed in pregnancy by increasing both food intake and body weight [117, 216, 223–225]. There is also evidence indicating that prolactin, and its related homologue PL, could contribute to the leptin-resistant state seen in pregnancy [116, 226]. More mechanistically, chronic intra-cerebral prolactin infusion prevents the decrease in body weight and food intake normally induced by leptin’s action on Pomc neurons [115, 226]. This absence of behavioral changes to leptin may be due to the reduction of STAT3 phosphorylation, a key factor in leptin signalling pathway [114, 116, 226] and could be linked with estrogen receptor co-transcription factors. Taken together, these data indicate that the early hyperphagia could be instated by the prolactin surges having orexigenic effects and maintained by the action of placental lactogens on prolactin receptors, thus inducing a leptin-resistant state.

**Fig. 2:**
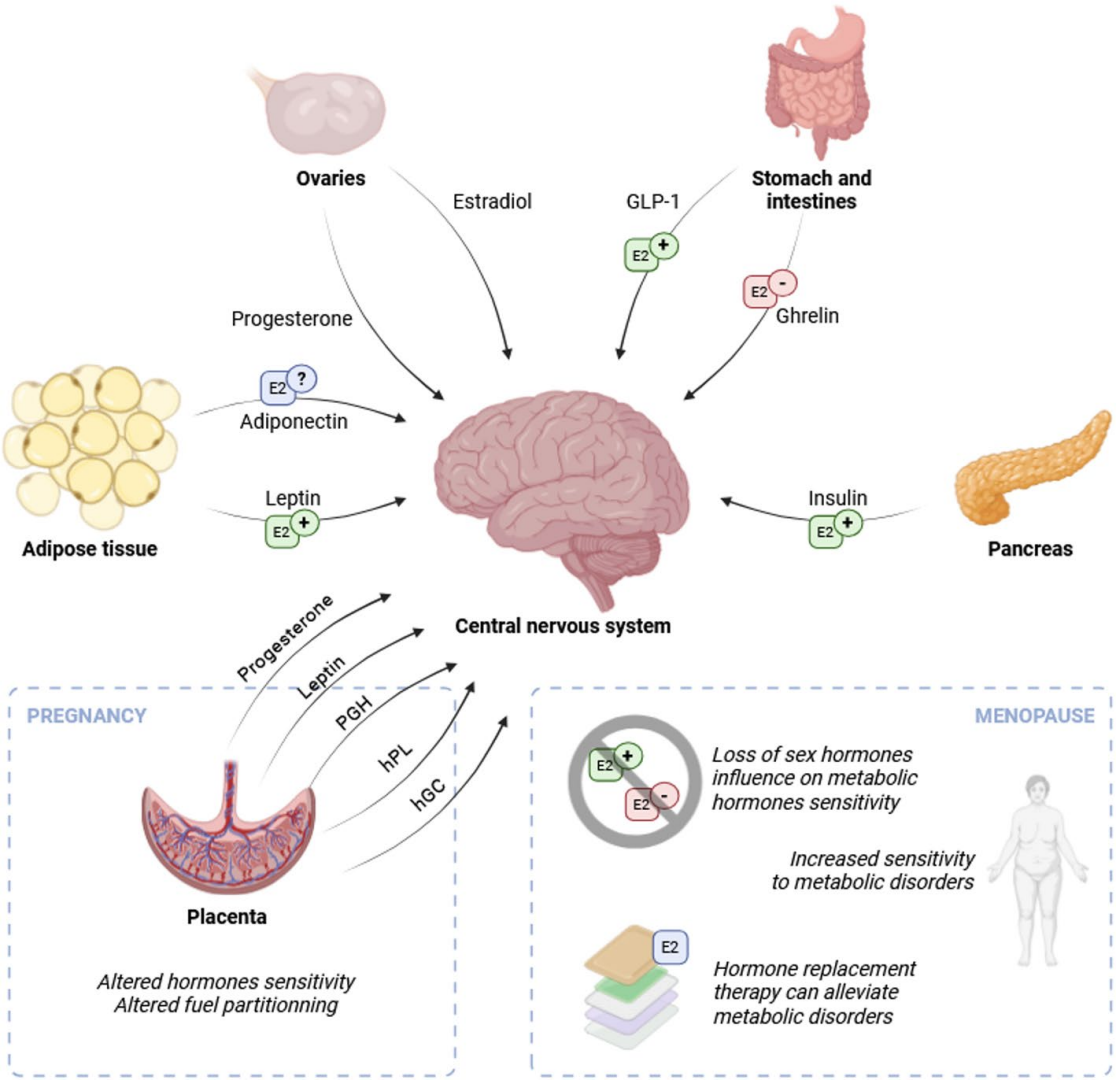
Hormonal interactions across the reproductive spectrum. Schematic representation of gonadal and metabolic hormones signaling to the central nervous system. Their synergistic effects with estradiol (E2) is highlighted via symbols on the respective arrows (E2+ for increased effects, E2– for decreased effects, E2? for unclear effects). Additional hormones signaling to the brain from the placenta are highlighted in the “Pregnancy” panel on the bottom left, while some hallmarks of “Menopause” are highlighted in the bottom right panel

## Hormonal synergies and current therapies

While not necessarily directly aiming to take advantage of hormonal synergies, multiple pharmacological treatments show metabolic benefits which are at least partly achieved via such interactions. Hormone replacement therapy for example, via oral or injectable estrogen preparations, is one the earliest medications formally approved by the FDA for the treatment of menopausal symptoms [227]. Studies have found that hormone replacement therapy can reduce abdominal fat and improve insulin resistance, although the effects are relatively modest [228, 229]. Tespria is another promising agonist of G protein-coupled estrogen receptor and was proven in preclinical studies to induce weight loss in postmenopausal mice, as well as in male mice [230].

Subcutaneous and oral GLP-1 receptor agonists (GLP-1RAs), in turn, offer a new option for weight management during menopause, as GLP-1RAs can still reduce weight in conditions of low estrogen levels [231]. Current evidence also supports that the dual GLP-1 and GIP agonist tirzepatide can significantly reduce weight in women during premenopause, perimenopause and postmenopause [232]. Whether these molecules and the new generation of polyagonists could see their weight loss and antidiabetic properties increased when combined with hormone replacement therapy is not clear yet. A recent study has shown that the weight loss response to semaglutide was improved in postmenopausal women under hormone replacement therapy, suggesting that taking advantage of hormonal synergies could increase the efficacy of incretin-based weight loss therapies [233]. These data go in line with the preclinical studies performed with a dual GLP-1-E2 agonist, which further ameliorates metabolic and cognitive health in rodent models [234, 235].

GDM, in turn, is approached very differently. The first-line treatment for GDM consists of lifestyle interventions, including controlled nutritional therapy, glucose monitoring, and moderate physical activity [236–239]. When glycemic targets are not achieved, second-line treatment with insulin or third-line treatment with oral antidiabetic agents such as glyburide or metformin is required to restore maternal glycemia [236, 240–243]. Insulin therapy effectively normalizes maternal and fetal glycemia [242, 243] and reduces the risk of pregnancy-related complications, including pre-eclampsia, in women unresponsive to lifestyle therapy [243, 244]. However, insulin-treated women must carefully balance insulin dosage, diet, and physical activity to prevent hypoglycemic episodes [243, 244].

Currently, GLP-1RAs are not approved for use during pregnancy due to limited human data [245]. Most available evidence arises from unknown exposure in women treated for metabolic conditions such as obesity prior to pregnancy recognition, resulting in early gestational exposure [245]. Although GLP-1RAs improve glycemic control and promote weight loss, making them a potential therapeutic option for women with obesity and diabetes before or during pregnancy [245], the use of GLP-1RAs is currently discouraged.

In animal models, prenatal exposure to GLP-1RAs has been associated with reduced fetal weight and growth [246, 247], delayed ossification, skeletal variants, and visceral abnormalities [248–251]. In contrast, human studies of first-trimester GLP-1RAs exposure have not identified increased rates or specific patterns of major congenital malformations compared with pregnancies in women with type 2 diabetes mellitus or obesity [245, 252, 253]. A case report of a woman with obesity and polycystic ovary syndrome exposed to exenatide prior to pregnancy recognition described one infant with a transient atrial septal defect that resolved spontaneously, while the second pregnancy was uncomplicated, and both children were healthy at follow-up [254]. Another study reported 1671 adverse outcomes associated with GLP-1RA exposure during pregnancy over a 20-year period, primarily affecting the reproductive and gastrointestinal systems, with spontaneous abortion and pre-eclampsia identified as the most serious outcomes [255]. Overall, the vast metabolic realignment, changes in hormones concentration, as well as modulation of hormones sensitivity, which all occur during pregnancy, complexify the use of incretin receptors agonists.

## Conclusion

In summary, the neuroendocrine interplay between sex hormones and metabolic hormones is both intricate and essential for maintaining energy homeostasis across the reproductive spectrum [summarized in Fig. 2]. Estradiol enhances the brain’s responsiveness to insulin, leptin, GLP-1, and ghrelin, often through synergistic pathways such as PI3K/Akt and JAK/STAT signaling and co-transcriptional mechanisms involving factors like Cited1. 

This synergy supports optimal regulation of appetite, energy expenditure, and glucose metabolism. However, the decline in estradiol during menopause disrupts this balance, leading to decreased hormone sensitivity, weight gain, insulin resistance, and metabolic disturbances—conditions often seen in postmenopausal women. During pregnancy, the addition of placental hormones further modulates this hormonal crosstalk, adapting hypothalamic control to accommodate shifting metabolic demands across trimesters.

Current anti-obesity and antidiabetic therapies are not specifically designed to take advantage of these synergies, although they may partly underlie their pharmacological success. Importantly, the role of estrogen receptor–associated co-transcription factors, such as Cited1 and PGC-1α, has emerged as a critical but still poorly understood component of this regulatory network. Unlocking the molecular roles of these co-regulators could provide new therapeutic avenues to restore metabolic balance, especially in contexts of hormonal transition such as menopause, or in contexts of inadequate hormonal sensitivity, such as in gestational diabetes and chronic obesity.

## Data Availability

No datasets were generated or analysed during the current study.
